# Quadrant Field Pupillometry Detects Melanopsin Dysfunction in Glaucoma Suspects and Early Glaucoma

**DOI:** 10.1038/srep33373

**Published:** 2016-09-13

**Authors:** Prakash Adhikari, Andrew J. Zele, Ravi Thomas, Beatrix Feigl

**Affiliations:** 1 Medical Retina and Visual Science Laboratories, Institute of Health and Biomedical Innovation, Queensland University of Technology, 60 Musk Avenue, Brisbane, QLD, 4059, Australia; 2 School of Optometry and Vision Science, Queensland University of Technology, Brisbane, QLD, Australia; 3 Queensland Eye Institute, South Brisbane, QLD, Australia; 4 University of Queensland, Brisbane, QLD, Australia; 5 School of Biomedical Sciences, Queensland University of Technology, Brisbane, QLD, Australia.

## Abstract

It is difficult to detect visual function deficits in patients at risk for glaucoma (glaucoma suspects) and at early disease stages with conventional ophthalmic tests such as perimetry. To this end, we introduce a novel quadrant field measure of the melanopsin retinal ganglion cell mediated pupil light response corresponding with typical glaucomatous arcuate visual field defects. The melanopsin-mediated post-illumination pupil response (PIPR) was measured in 46 patients with different stages of glaucoma including glaucoma suspects and compared to a healthy group of 21 participants with no disease. We demonstrate that the superonasal quadrant PIPR differentiated glaucoma suspects and early glaucoma patients from controls with fair (AUC = 0.74) and excellent (AUC = 0.94) diagnostic accuracy, respectively. The superonasal PIPR provides a linear functional correlate of structural retinal nerve fibre thinning in glaucoma suspects and early glaucoma patients. This first report that quadrant PIPR stimulation detects melanopsin dysfunction in patients with early glaucoma and at pre-perimetric stages may have future implications in treatment decisions of glaucoma suspects.

Primary open-angle glaucoma (POAG) is a leading cause of irreversible blindness^1^. It causes a progressive and chronic loss of Retinal Ganglion Cells (RGCs) and their axons, leading to optic nerve atrophy^2^. Standard Automated Perimetry (SAP) is the principle measure of glaucomatous visual deficits and the visual field loss correlates with regional RGC loss^3^,^4^,^5^, but significant RGC damage occurs before a visual defect is detected^3^,^6^,^7^. Retinal Nerve Fibre Layer (RNFL) imaging can potentially detect early structural changes in glaucoma^8^ that correspond with sectoral visual field deficits^7^,^9^. Together, the visual fields and RNFL imaging provide evidence for a preferential vulnerability of the inferior RNFL in glaucoma^8^,^10^,^11^. While the detection of early glaucomatous damage using emerging technologies shows promising results^8^,^11^,^12^,^13^,^14^,^15^,^16^,^17^,^18^,^19^, the detection of pre-perimetric glaucomatous deficits still remains a challenge.

The discovery of melanopsin^20^,^21^,^22^ expressing intrinsically photosensitive Retinal Ganglion Cells (ipRGCs) adds a new dimension to the detection and monitoring of the progression of retinal and optic nerve disorders, including glaucoma through pupillometry^23^,^24^,^25^,^26^,^27^,^28^,^29^,^30^,^31^,^32^,^33^,^34^,^35^,^36^ (see Feigl & Zele, 2014 for review^32^). Five different ipRGC subtypes in transgenic mice and two ipRGC subtypes in primates have been identified that differ in morphology and project to different brain areas^32^,^37^. The main subtype of interest for this study is the M1 ipRGC which pre-dominantly innervates the olivary pretectal nucleus shell for pupil control^38^. These inner retinal photoreceptors entirely drive the post-illumination pupil response (PIPR)^21^,^28^,^39^. This sustained pupil constriction after light offset matches the spectral sensitivity of the melanopsin pigment (≥1.7 s after light offset) such that it can be used as a direct biomarker of ipRGC function^21^,^28^,^39^,^40^. The pupil light reflex (PLR) during light stimulation is mediated via both outer retinal and inner retinal photoreception with the relative photoreceptor contributions depending on the stimulus paradigm^41^,^42^,^43^.

Melanopsin function in glaucoma has been assessed by measuring the PLR during light stimulation^27^,^35^,^44^ and the PIPR after light offset^23^,^24^,^25^,^26^,^36^,^45^. Focal retinal stimulation pupillometry^23^,^28^,^35^,^44^,^46^,^47^,^48^,^49^,^50^ is useful for detecting localised damage in ocular diseases including glaucoma. In late, but not early glaucoma, there is a relative afferent pupillary defect in the quadrant field^44^,^48^ and localised changes in the PLR are detectable with multifocal stimuli^35^. The melanopsin-mediated PIPR is affected in the central visual field in late glaucoma^23^,^24^,^25^,^26^,^36^,^45^, but not in early glaucoma^23^. A recent study observed a normal PIPR in ocular hypertension^45^. The PIPR has not been measured in glaucoma suspects. Based on typical glaucomatous arcuate deficits^51^,^52^ and RNFL defects^8^,^11^, and evidence that regional visual field deficits can be mapped to sectoral optic disc abnormalities in glaucoma^7^,^9^,^53^,^54^, we introduce a quadrant field stimulation paradigm using optimised pupillometry protocols^39^ in order to differentiate melanopsin function in glaucoma suspects and manifest glaucoma at different severity stages from healthy eyes by measuring the PLR and PIPR. Based on evidence that melanopsin dysfunction is related to sleep disorders in late glaucoma patients^26^ and reports that melanopsin gene (*OPN4*) variants modulate the pupil response and sleep behaviour^55^,^56^,^57^,^58^, a secondary aim was to investigate if the established *OPN4* variants could affect the PIPR or sleep, independent of the different stages of glaucoma.

## Methods

### Participants

Patients were recruited from the private practice of one glaucoma specialist who determined the stage of glaucoma (suspect, early, moderate, advanced). The diagnosis of glaucoma suspect and glaucoma followed the American Academy of Ophthalmology Preferred Practice Pattern Guidelines^2^,^59^. The diagnosis of POAG was based on the presence of a combination of glaucomatous optic disc (diffuse or focal narrowing of the rim, rim notching defined as one clock hour of rim loss at the inferior or superior quadrants, disc haemorrhage, rim to disc ratio <0.1^60^, diffuse or focal nerve fibre layer damage, cup disc ratio >0.7, inter-eye asymmetry of cup disc ratio >0.2) with confirmed, correlating, and repeatable visual field defects on standard automated perimetry (SAP). Primary open-angle glaucoma was classified as early (mean deviation; MD < −6 dB), moderate (−6 dB ≤ MD < −12 dB), and advanced (MD > −12 dB) on the basis of a visual field mean deviation according to the Hodapp, Parrish, and Anderson classification^61^. Glaucoma suspects were defined on the basis of the optic nerve changes described above, but no visual field defects that correlated with the clinical examination of the optic disc^59^.

We recruited 67 participants in the study: 34 patients with early (n = 22), moderate (n = 6), and advanced (n = 6) POAG (age range = 50–90 years), 12 glaucoma suspects (age range = 50–77 years), and 21 healthy controls (age range = 42–74 years) (see ‘Results’ for participant characteristics). As moderate and advanced glaucoma patients are known to have a reduced PIPR^23^ (and pupillometry is not needed for further differentiation), they were analysed together as the “late” glaucoma group (n = 12). Based on a previous study^23^, a sample size calculation determined that 12 participants in each sub-group are required to achieve 90% power (effect size = 1.29) (G*Power 3.1) for detecting a significant mean difference of 5.8% in the PIPR amplitude between glaucoma suspects/patients and healthy controls.

All glaucoma suspects and patients underwent a complete eye examination including visual acuity, intraocular pressure (IOP, Goldmann tonometer AT 900, Haag-Streit AG, Koeniz, Switzerland), colour vision (Ishihara), slit lamp biomicroscopy, ophthalmoscopy, visual field (Humphrey 30-2, Humphrey Field Analyzer, HFA, Carl Zeiss Meditec, Inc. Dublin, CA), and optical coherence tomography (OCT) nerve fibre layer and disc map (Cirrus-HD OCT, Carl Zeiss Meditec, Inc. Dublin, CA). All glaucoma suspects and patients were using IOP lowering topical medications; there is a known miotic effect from brimonidine^62^,^63^,^64^,^65^ and travoprost^66^, whereas bimatoprost^67^, brinzolamide^68^,^69^, latanoprost^70^, and timolol^70^,^71^,^72^ have minimal (≤0.2 mm) or no miotic effects on the pupil. The potential effect of any drug mediated miosis on the pupil light reflex amplitude was compensated for by normalising the amplitude to the baseline pupil diameter^73^,^74^. Nineteen patients with manifest glaucoma had undergone some kind of surgical intervention for glaucoma (trabeculectomy (n = 2), selective laser trabeculoplasty (n = 7), and laser peripheral iridotomy (n = 10)); the iris dynamics (PIPR redilation velocity) used for determining the PIPR amplitude did not differ between patients with and without surgery. There is evidence that the PIPR amplitude increases after cataract surgery due to an increased retinal irradiance that enhances ipRGC photoreception^75^. A small proportion of our participants in each group (one control, two suspects, five early, and four late glaucoma patients) had cataract surgery with intraocular lens implants (see ‘Results’). They had normal pupillary margins and no sphincter tear and the PLR and PIPR amplitudes were similar to the fellow participants with natural lenses indicating cataract surgery had limited or no effect on the pupil results. Participants with ocular pathology other than glaucoma were excluded, including any kind of retinopathy or optic neuropathy as well as corneal opacities, lenticular opacification > grade 2 (Lens Opacities Classification System, LOCS III)^76^, and a history of uveitis.

The healthy controls were recruited from a university cohort and age-matched with the glaucoma suspects because the primary aim of the study was to detect early melanopsin dysfunction in suspects. They had no ocular or systemic pathology, no corneal opacity, lenticular opacification < grade 2 (LOCS III), and no history of uveitis. Because the PIPR is robust to healthy ageing^74^,^77^, exact age matching is not mandatory but was performed to enable comparisons with other studies. Absence of ocular pathology was confirmed with a complete eye examination as detailed above and including contrast-sensitivity (Pelli-Robson Chart) and colour vision (Lanthony Desaturated D-15 test).

Sleep quality was assessed using the Pittsburgh Sleep Quality Index (PSQI)^78^. DNA genotyping was performed to detect the *OPN4* gene single nucleotide polymorphisms (SNPs) P10L and I394T; genotyping followed established procedures and as previously performed in our studies^79^,^80^.

All experimental protocols were approved by the Queensland University of Technology (QUT) Human Research Ethics Committee (approval no.: 1400000793) and conducted in accordance with their guidelines. The research followed the tenets of the Declaration of Helsinki and informed consent was obtained from all participants.

### Pupillometry

**Figure 1:**
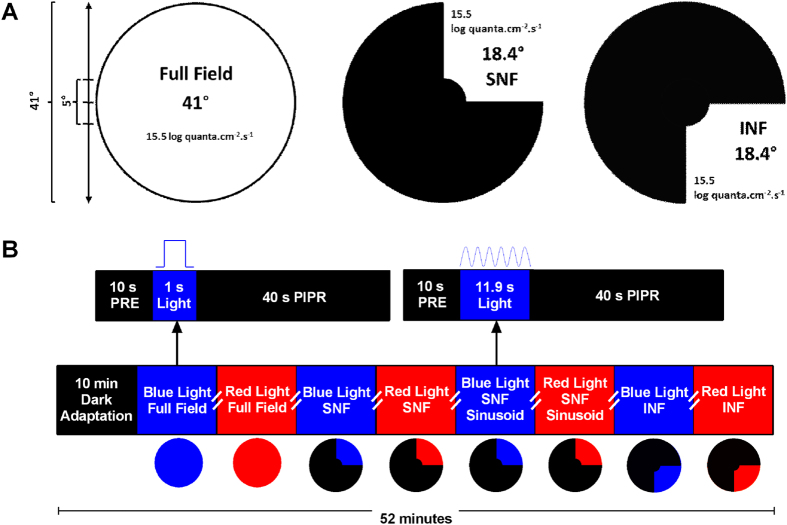
Characteristics of the pupillometry stimulus fields represented in the visual space of the left (test) eye (Panel A). Schematic of the pupillometry protocol (Panel B). The pulse and sinusoidal stimulus protocols indicated by arrows were common for blue and red stimuli. Blue stimuli (blue rectangles) and red stimuli (red rectangles) were alternated and measurements were repeated twice. The double slashes indicate a two-minute interval between the tests to allow the pupil return to the baseline size^39^. PRE, pre-stimulus; PIPR, post-illumination pupil response; SNF, superonasal field; INF, inferonasal field.

We designed two new pupillometric stimulus paradigms based on the location of Retinal Nerve Fibre Layer (RNFL) defects in glaucoma^8^,^11^,^51^,^52^: A superonasal field (SNF, 18.4°, retinal image altitude: 8 mm) and an inferonasal field (INF, 18.4°, retinal image altitude: 8 mm) stimulus, both sparing the central 5° (retinal diameter: 2 mm) to avoid the stimulation of the foveal zone devoid of ipRGCs based on their anatomical distributions^81^,^82^ (Fig. 1A). A conventional full field stimulus (41° in diameter, retinal image diameter: 17.9 mm) was also applied. The PLR was measured in Maxwellian view with a custom-designed pupillometer (see Feigl *et al*.^23^ and Adhikari *et al*.^39^ for details) using 1 s light pulses based on our previous research where 1 s pulses produced larger PIPRs than longer (10 s and 30 s) stimulus durations^39^. The stimuli included narrow-band blue lights (short wavelength, λ_max_ = 464 nm, 20 nm band-width at half maximum, corneal irradiance: 15.5 log quanta.cm^−2^ s^−1^, luminance: 2.9 log cd.m^−2^) with high melanopsin excitation (8601.7 α-opic lux^83^) and red lights (long wavelength, λ_max_ = 658 nm, 22 nm band-width at half maximum, corneal irradiance: 15.5 log quanta.cm^−2^ s^−1^, luminance: 3.1 log cd.m^−2^) with low melanopsin excitation (0.5 α-opic lux); the presentation order was alternated to account for the effect of a suggested melanopsin bistability^84^. In addition to the 1 s pulse, the SNF stimulus was also presented as a sinusoidal stimulus (0.5 Hz, 6 cycles, 11.9 s duration) to evaluate the interaction between inner and outer retinal inputs to the phasic pupil response^32^,^34^,^85^ (Fig. 1B).

In all participants, the left pupil was dilated (1% Tropicamide, Minims, Chauvin Pharmaceuticals Ltd., Romford, UK) to maintain a constant retinal irradiance and was stimulated to measure the consensual PLR of the right eye. All measurements were preceded by 10 minutes dark adaptation (<1 lux) to eliminate the effect of prior light exposure on the pupil light response^39^. The baseline pupil diameter was measured in the dark for 10 s before stimulus onset and the PIPR was measured for 40 s after stimulus offset. Measurements were repeated twice; the intra-individual coefficients of variation (CV) of the peak pupil constriction and PIPR amplitudes with blue full field stimuli were 0.03 ± 0.03 and 0.10 ± 0.11 (mean ± SD), respectively, which are below the recommended acceptance limit for CV^86^. Pupillometry was performed between 10 AM and 5 PM to minimise the effects of circadian variation of the PIPR amplitude^46^,^87^.

### Quantification of the PLR and PIPR

The PLR during light stimulation was quantified using the transient PLR, peak pupil constriction amplitude, time to peak constriction, and phase amplitude percentage (PAP) metrics defined in Table 1. The PIPR amplitude was quantified with the 6 s and plateau PIPR metrics based on previous findings that determined optimum PIPR metrics (Adhikari *et al*.^39^). Since both the plateau and 6 s PIPR metrics showed similar outcomes, only the 6 s PIPR is presented and subsequently reported as the PIPR amplitude.

### Statistical analysis

GraphPad Prism (GraphPad Software, Inc., CA, USA) was used for statistical analysis. Comparisons of the PLR and PIPR metrics were performed between the four groups: Healthy controls, glaucoma suspects, early glaucoma, and late glaucoma. The data frequency distributions were evaluated with the D’Agostino and Pearson omnibus normality test. Normal data were analysed with one-way ANOVA (Tukey’s multiple comparisons) and non-normal data were analysed with the Kruskal-Wallis test (Dunn’s multiple comparisons) to compare the PLR and PIPR between the four (suspect, early, late, and control) study groups (95% confidence interval, p < 0.05, Geisser-Greenhouse correction). The pupil metrics are presented in Box-and-Whisker plots showing the median, 25% and 75% quartiles, and range of the data. The relationship between the PIPR amplitude and mean RNFL thickness or visual field MD was evaluated with linear or non-linear regression, respectively and the statistical significance of linear regression was determined on the basis of whether or not the slope of the best-fitting regression line was significantly different from zero using F-test (95% confidence interval, p < 0.05). Receiver operating characteristic (ROC) analysis was performed to determine the diagnostic accuracy of the PIPR to differentiate glaucoma suspects and established glaucoma patients from healthy control participants.

## Results

**Figure 2:**
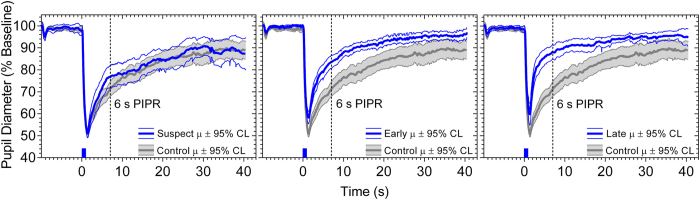
Averaged pupil traces for controls (n = 21), glaucoma suspects (n = 12), early glaucoma (n = 22) and late glaucoma patients (n = 12) in response to a 1 s, 464 nm, 15.5 log quanta.cm^−2 ^s^−1^ light pulse (indicated by the blue bar on the abscissa at 0 s) presented in the superonasal quadrant field. The shaded areas for the controls and thinner lines for the glaucoma suspects, early glaucoma and late glaucoma patients indicate 95% confidence limits (CL) of the mean (μ). The vertical line denotes the PIPR amplitude.

**Figure 3:**
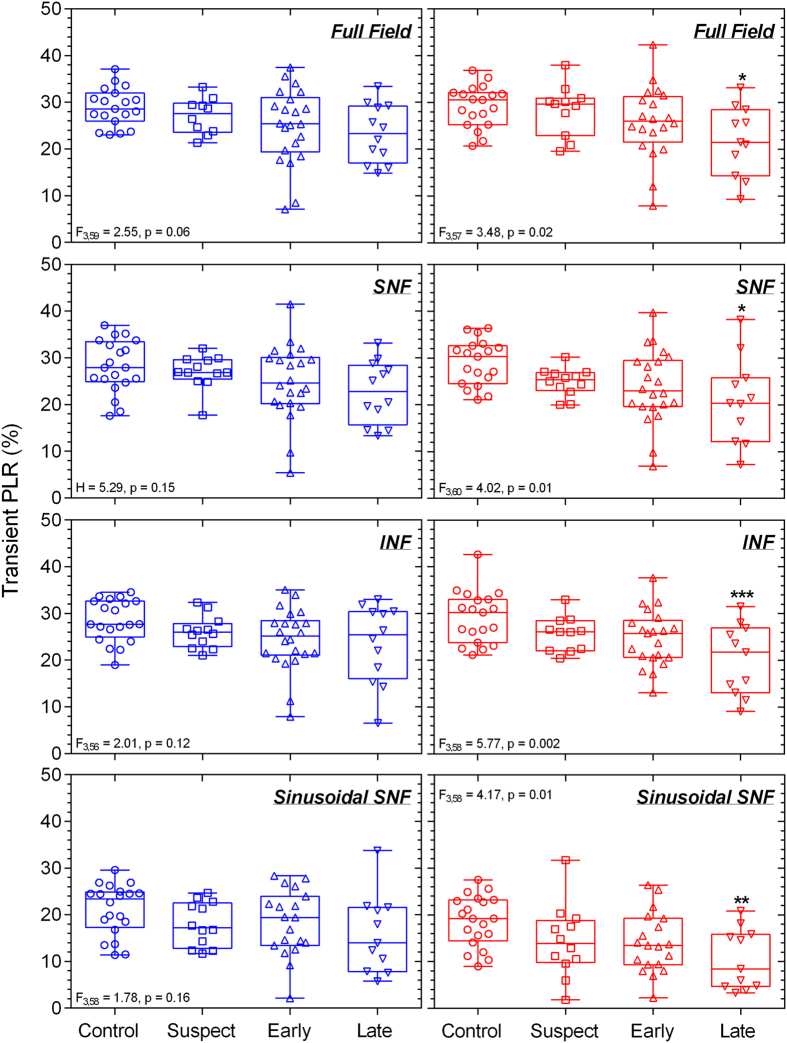
The transient pupil light reflex (PLR) plotted as a function of percentage (%) baseline pupil diameter with different stimulus field conditions (full field, SNF, INF) for controls (n = 21), glaucoma suspects (n = 12), early glaucoma patients (n = 22), and late glaucoma patients (n = 12). Left panels show the data for the blue stimulus lights; right panels show the data for the red stimulus lights. A significant reduction is demonstrated in response to red stimuli in late glaucoma for all field sizes. Asterisks indicate statistically significant difference from controls (*p < 0.05, **p < 0.01, ***p < 0.001). SNF, superonasal field; INF, inferonasal field.

**Figure 4:**
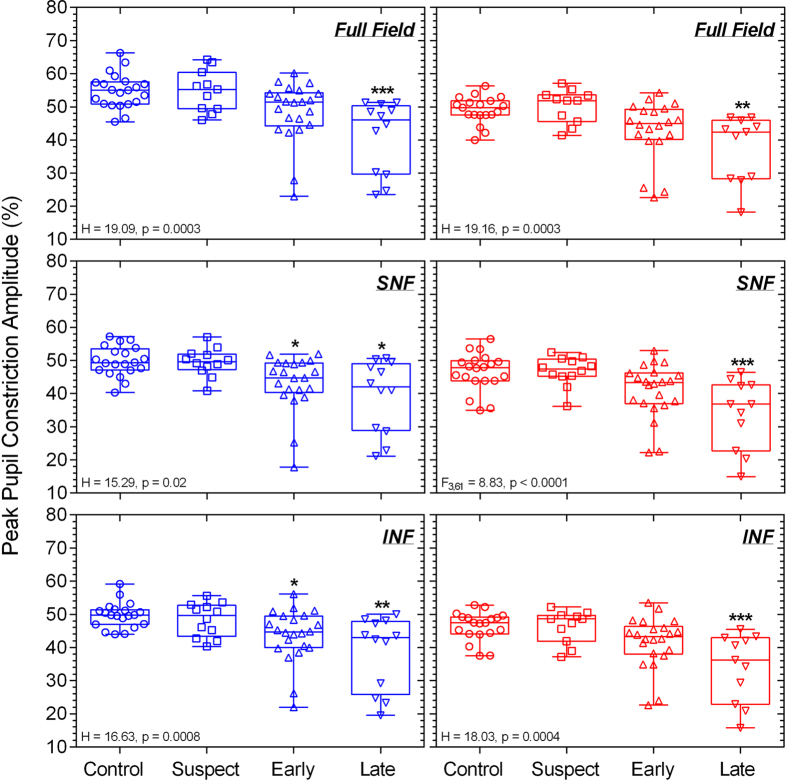
The peak pupil constriction amplitude plotted as a function of percentage (%) baseline pupil diameter with different stimulus field conditions (full field, SNF, INF) for controls (n = 21), glaucoma suspects (n = 12), early glaucoma patients (n = 22), and late glaucoma patients (n = 12). Left panels show the data for the blue stimulus lights; right panels show the data for the red stimulus lights. A significant reduction in amplitude is evident in response to red and blue lights in late glaucoma for all field sizes and for blue lights also in early glaucoma for both quadrant stimuli. Asterisks indicate statistically significant difference from controls (*p < 0.05, **p < 0.01, ***p < 0.001). SNF, superonasal field; INF, inferonasal field.

**Figure 5:**
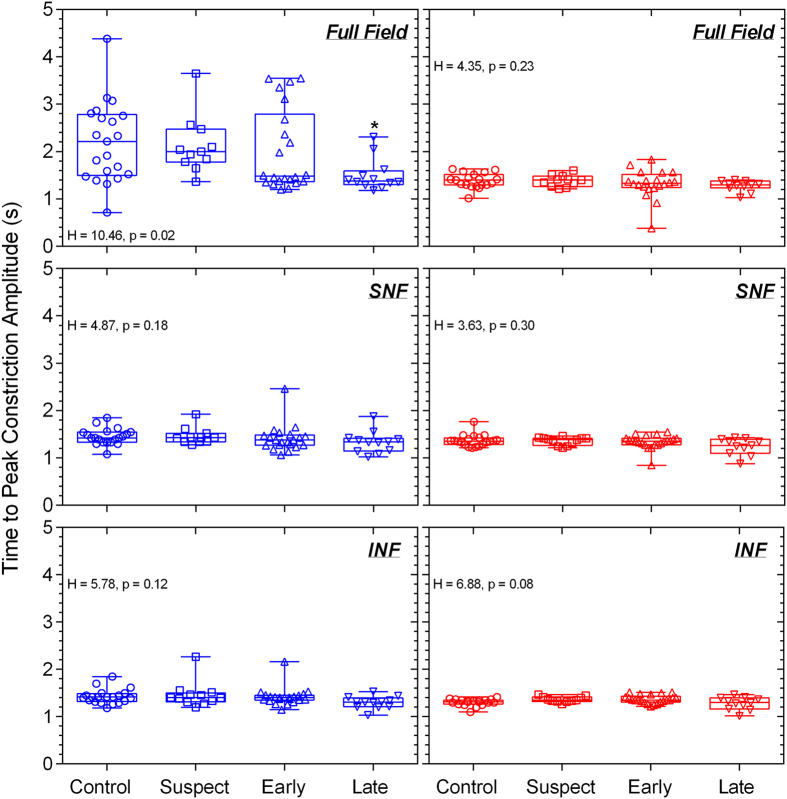
The time to peak pupil constriction amplitude in seconds with different stimulus field conditions (full field, SNF, INF) for controls (n = 21), glaucoma suspects (n = 12), early glaucoma patients (n = 22), and late glaucoma patients (n = 12). Left panels show the data for the blue stimulus lights; right panels show the data for the red stimulus lights. A significantly shorter time to the peak constriction is demonstrated in response to blue lights in late glaucoma for the full field stimulus only. Asterisks indicate statistically significant difference from controls (*p < 0.05). SNF, superonasal field; INF, inferonasal field.

**Figure 6:**
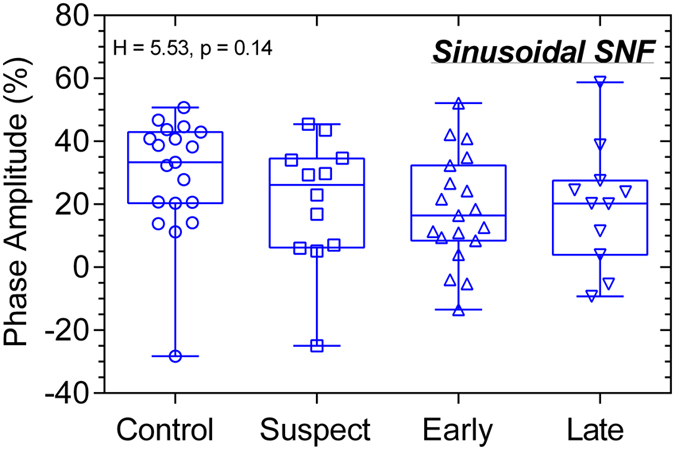
The phase amplitude percentage (PAP) with sinusoidal superonasal field (SNF) stimuli shows no significant difference between controls (n = 21), glaucoma suspects (n = 12), early glaucoma patients (n = 22), and late glaucoma patients (n = 12).

The clinical characteristics of the 67 participants given in Table 2 indicate that RNFL thickness and visual field sensitivity were significantly reduced in early and late glaucoma patients compared to controls. The results of the statistical analyses are included within the Figures. The averaged pupil response traces to the blue stimulus with high melanopsin excitation for the controls, glaucoma suspects, early glaucoma and late glaucoma participants (Fig. 2) show that the mean peak pupil constriction and the PIPR amplitudes differed between the groups; detailed results follow. The transient PLR (Fig. 3) to the 1 s red stimuli was significantly reduced in late glaucoma for all field sizes (full field: F_3,57_ = 3.48, p = 0.02; SNF: F_3,60_ = 4.02, p = 0.01; INF: F_3,58_ = 5.77, p = 0.002) when compared to healthy controls, and with the SNF sinusoidal stimulus, but not for the blue stimulus. The peak pupil constriction amplitude (Fig. 4) was significantly reduced in late glaucoma compared to controls with blue (full field: H = 19.09, p = 0.0003; SNF: H = 15.29, p = 0.02; INF: H = 16.63, p = 0.0008) and red (full field: H = 19.16, p = 0.0003; SNF: F_3,61_ = 8.83, p < 0.0001; INF: H = 18.03, p = 0.0004) stimuli for all stimulus fields. With the blue quadrant stimuli, the peak constriction was also significantly reduced in early glaucoma compared to controls. The time to peak pupil constriction (Fig. 5) was significantly shorter in late glaucoma compared to controls with the blue full field stimulus (H = 10.46, p = 0.02). The phase amplitude percentage (PAP) derived from the SNF sinusoidal stimulation was not significantly different between the four participant groups (Fig. 6).

**Figure 7:**
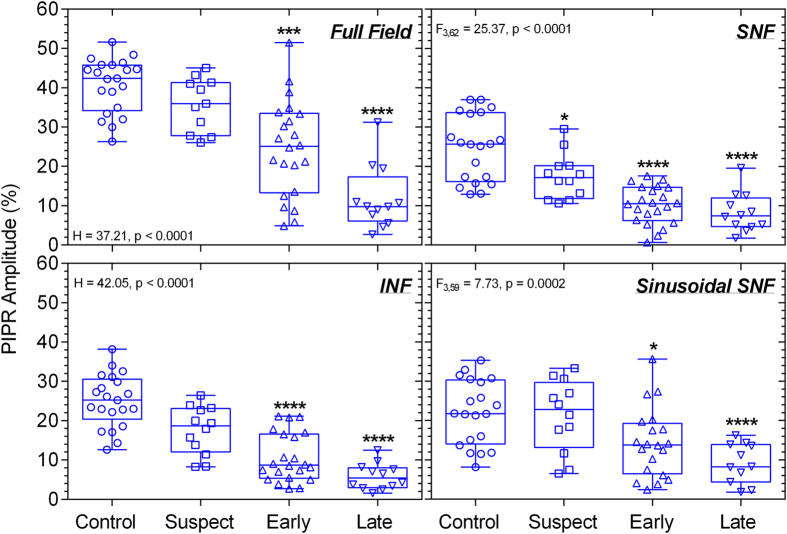
The post-illumination pupil response (PIPR) amplitude plotted as a function of percentage (%) baseline pupil diameter with different stimulus field conditions (full field, SNF, INF) for controls (n = 21), glaucoma suspects (n = 12), early glaucoma patients (n = 22), and late glaucoma patients (n = 12) shows deficits for all field sizes in early and late glaucoma. Glaucoma suspects exhibit superonasal deficits and differ significantly from early glaucoma patients. Asterisks indicate statistically significant difference from controls (*p < 0.05, ***p < 0.001, ****p < 0.0001). SNF, superonasal field; INF, inferonasal field.

**Figure 8:**
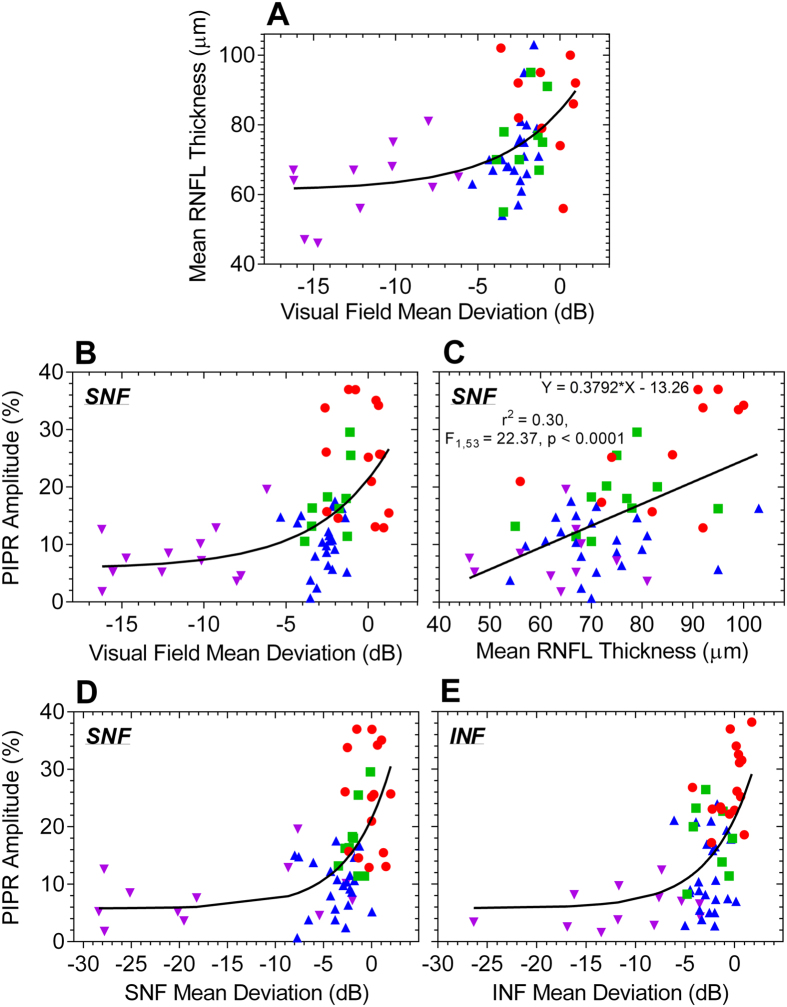
Scatterplots of mean retinal nerve fibre layer (RNFL) thickness versus visual field mean deviation (MD) (**A**), the post-illumination pupil response (PIPR) amplitude versus visual field MD (**B**), the PIPR versus mean RNFL thickness (**C**), the superonasal field (SNF) PIPR versus superonasal visual field MD (**D**), and the inferonasal field (INF) PIPR versus inferonasal visual field MD (**E**) in controls (red circles), glaucoma suspects (green squares), early glaucoma patients (blue triangles), and late glaucoma patients (purple inverted triangles). Visual field MD has a non-linear relationship with RNFL thickness and the PIPR; and lower PIPRs are linearly related to reduced RNFL thickness. Solid lines are the best fitting models.

The melanopsin-controlled PIPR was significantly reduced in early and late glaucoma patients compared to controls with the quadrant (SNF: F_3,62_ = 25.37, p < 0.0001; INF: H = 42.05, p < 0.0001) and full field stimulation (H = 37.21, p < 0.0001) (Fig. 7). Importantly, glaucoma suspects exhibited significantly reduced PIPR amplitudes with SNF stimuli, indicative of the effectiveness of the quadrant field paradigm to detect melanopsin dysfunction. Given that the superonasal field PIPR amplitude differentiated melanopsin dysfunction in glaucoma suspects from controls, we further compared the superonasal field results with SAP visual fields and OCT (RNFL thickness) used in conventional glaucoma screening and monitoring. A non-linear model^7^ best described the relationships between visual field MD and mean RNFL thickness, and with PIPR amplitude (Fig. 8A,B). The superonasal and inferonasal visual field MD also showed non-linear relationships with the corresponding superonasal and inferonasal field PIPR amplitude (Fig. 8D,E). Notably, a linear model best described the positive association between mean RNFL thickness and the PIPR amplitude (Fig. 8C)^88^. There was no association between IOP and the PIPR.

**Figure 9:**
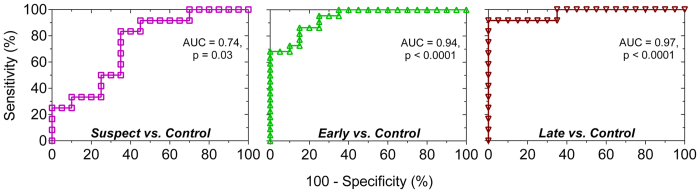
Receiver operating characteristic (ROC) curves for the post-illumination pupil response (PIPR) amplitude with blue superonasal field (SNF) stimuli demonstrate fair to excellent diagnostic accuracy of the PIPR to differentiate glaucoma suspects, early glaucoma, and late glaucoma patients from controls.

The ROC analysis demonstrated that the superonasal PIPR has a fair diagnostic accuracy^89^ (AUC = 0.74, p = 0.03) to discriminate melanopsin dysfunction in glaucoma suspects from healthy eyes and excellent diagnostic accuracy to discriminate early glaucoma (AUC = 0.94, p < 0.0001) and late glaucoma (AUC = 0.97, p < 0.0001) from healthy eyes (Fig. 9). Based on the ROC analysis, PIPR cut-off values were chosen to provide sensitivities >90% to detect melanopsin dysfunction in glaucoma suspects and patients compared to controls and the corresponding positive and negative likelihood ratios (LR’s) were calculated. At the selected PIPR cut-off values for glaucoma suspects and patients (Table 3), the positive LR’s signify ability to cause small to moderate increases in the clinical probability of glaucoma whereas the corresponding negative LR’s signify ability to cause moderate to large decreases in the clinical probability of glaucoma^89^,^90^. For glaucoma suspects, a 25.6% PIPR cut-off value had a positive LR of 2.0 and a negative LR of 0.15; for early glaucoma, a 17.0% PIPR had a positive LR of 3.8 and a negative LR of 0.06. In late glaucoma, a 13.8% PIPR had a positive LR of 9.2 and a negative LR of 0.09. The PIPR values below the cut-off generally resulted in decreased sensitivities and increased specificities and likelihood ratios whereas the PIPR values above the cut-off resulted in increased sensitivities and decreased specificities and likelihood ratios as might be expected.

**Figure 10:**
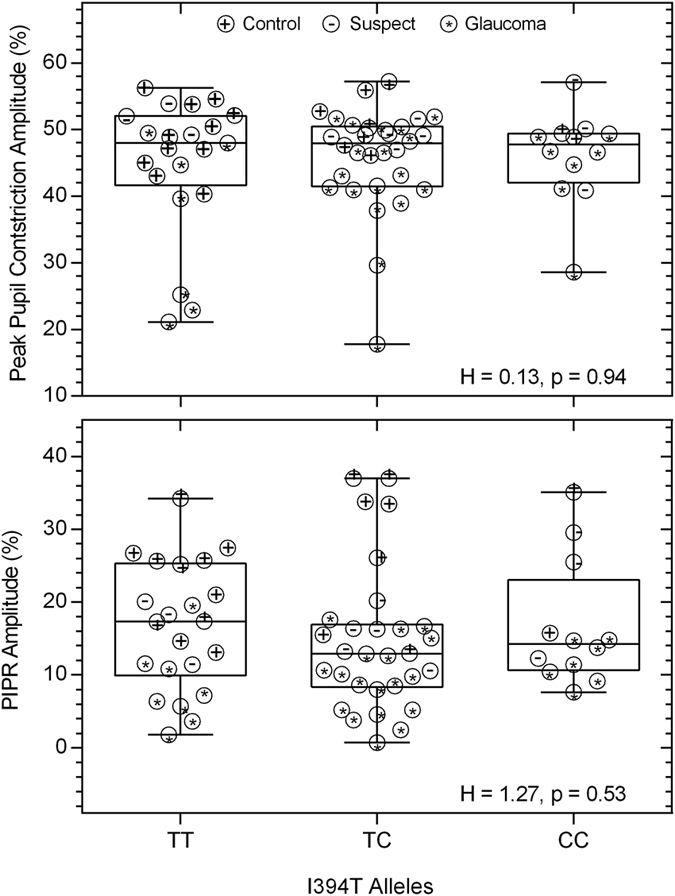
The peak pupil constriction amplitude and the post-illumination pupil response (PIPR) amplitude plotted as a function of percentage (%) baseline pupil diameter in participants with the TT, TC, and CC alleles of the *OPN4* gene single nucleotide polymorphism I394T.

All participants had normal sleep patterns. However, the TT risk allele of the *OPN4* SNP P10L that has been associated with sleep disturbances was present in only one participant (an early glaucoma patient). The peak pupil constriction amplitude and the PIPR amplitude did not vary significantly within the *OPN4* SNP I394T alleles that have been previously linked with lower PIPR amplitudes (Fig. 10).

## Discussion

Our results show that selective superonasal quadrant field stimulation can be used to detect melanopsin expressing intrinsically photosensitive Retinal Ganglion Cell (ipRGC) dysfunction in glaucoma suspects in the absence of perimetric deficits (Fig. 7). Importantly, this new PIPR quadrant stimulation paradigm can differentiate melanopsin dysfunction in glaucoma suspects and early glaucoma from healthy controls with fair and excellent diagnostic accuracy, respectively (Fig. 9). In contrast, full field pupillometry stimulation is sensitive only to changes in the melanopsin-mediated PIPR in more advanced stages of glaucoma^23^,^24^. The negative likelihood ratios in glaucoma suspects and early glaucoma were estimated using cut-off values to provide sensitivities >90% and indicate that the PIPR quadrant test may have potential in the important clinical decision to rule out disease and to estimate the required frequency of follow up for the individual patient^89^.

Our finding of reduced PIPR amplitudes with selective superonasal field stimulation in glaucoma suspects and early glaucoma is consistent with rodent models of ipRGC abnormalities in the early stages of experimental glaucoma^91^,^92^,^93^,^94^,^95^,^96^ and with the preferential vulnerability of the inferior retina in glaucoma in humans^8^,^10^,^11^. Reduced blood flow resulting from high IOP or reduced arterial pressure can lead to ischaemic nerve fibre damage in glaucoma^97^ which is most pronounced in the inferior retina^10^. The inferotemporal RNFL loss is most frequently detected in the OCT in glaucoma suspects and early glaucoma patients and has the highest diagnostic accuracy for early glaucoma^8^,^11^. Additionally, the linear relationship between a lower PIPR amplitude and a reduced mean RNFL thickness in our study sample (Fig. 8C) is in agreement with recent studies^27^,^88^. The linear relationship between the PIPR and RNFL thickness may therefore have value in the prediction of functional deficits based on structural defects.

Advanced glaucoma patients with deficits in the central 7° on microperimetry that are not evident on Standard Automated Perimetry (SAP), have greater PIPR deficits compared to early glaucoma patients^23^. This evidence of ipRGC dysfunction in the central retina^23^ and the association between a lower PIPR and greater visual field defects in advanced glaucoma^24^ highlight the importance of developing and refining selective field stimulation pupillometry.

The reduced transient PLR to red stimuli in late glaucoma (Fig. 3) indicates cone dysfunction^43^ consistent with evidence of a decrease in cone density^98^ as demonstrated by a reduced photopic a-wave amplitude in the electroretinogram in glaucoma patients^99^. Though rod loss and dysfunction have been reported in glaucoma^100^,^101^, our pupillometry protocol was optimised to measure ipRGC function rather than rod function. The stimulus protocol may reveal rod deficits in glaucoma when tested at scotopic light levels^28^,^43^.

The peak pupil constriction amplitude (Fig. 4) was reduced in late glaucoma in agreement with the literature^27^,^44^,^102^,^103^. With high irradiance short wavelength lights, the peak pupil constriction amplitude quantifies both outer and inner retinal contributions to the PLR^41^ and our finding indicates that these contributions are compromised in late glaucoma. In this study, a deficit in the peak constriction amplitude became evident in early glaucoma only with the quadrant field blue stimulus, and not with the full field, possibly due to the signals from the intact outer retina photoreceptors masking the presence of localised ipRGC dysfunction. The time to peak constriction with the full field stimulus (Fig. 5) was shorter in late glaucoma compared to healthy eyes, likely due to ipRGC dysfunction. The ipRGC inputs to the pupil control pathway has larger spatial summation areas^49^,^50^ compared to the spatial summation areas of image forming vision^104^ and therefore with high irradiance short wavelength lights, ipRGCs produce larger constriction amplitudes and delay the time to peak constriction^39^ compared to rods and cones^105^. Based on this evidence, we infer that the shorter time to peak constriction in late glaucoma with full field short wavelength stimuli is likely due to ipRGC dysfunction. We also determined that the inner and outer retinal interactions quantified with the phase amplitude percentage (PAP) are not affected in glaucoma (Fig. 6).

Our participants did not exhibit sleep disorders as assessed with the PSQI; it is possible that sleep disorders may have been detected with polysomnography as previously reported^26^ but this was outside scope of our study. While the *OPN4* SNP P10L TT genotype has been demonstrated to be associated with poorer sleep quality, there was only one patient with the TT risk allele in our cohort. We therefore infer that our results of normal sleep behaviour in glaucoma patients may in some part reflect the low frequency of the risk allele in our participants and the smaller sample of late glaucoma patients. Notably, the I394T genotype did not affect any of the pupil metrics (Fig. 10), suggesting that this *OPN4* gene variant was not a contributor to the lower PIPR and PLR responses in the glaucoma patients.

Increased PIPR amplitudes have been found to occur in older people due to lens scattering^106^, however we limited lens scattering by excluding patients with lens opacification >2 (LOCS III). Based on literature evidence, the PIPR is robust to healthy ageing in humans^74^,^77^ and ipRGC density is independent of age in rodent models^107^, suggesting that exact age matching is not mandatory. However, if lens scatter had affected the PIPR, higher, and not lower PIPR amplitudes as found in this study, might have been observed.

The primary aim of this study was to determine if the quadrant pupillometry protocol can detect a mean difference in melanopsin cell function in glaucoma suspects, early glaucoma patients, and controls; the sample size was therefore optimised to examine this aim rather than to determine the diagnostic accuracy and as such the reported sensitivity and specificity of the PIPR have wide confidence limits (Table 3). The ROC AUC of 74% in glaucoma suspects is not optimal; studies with larger samples are necessary to refine our estimate of the diagnostic accuracy of the quadrant pupillometry protocol and a longitudinal study is needed to determine if suspects proceed to manifest glaucoma. Nevertheless, the reported point estimates and upper end of the confidence limits suggest that the test may have good potential as a clinical tool in the detection of pre-perimetric glaucomatous damage and the identification of early glaucoma.

In conclusion, we show that the superonasal field melanopsin PIPR measurement can detect inner retinal melanopsin dysfunction in glaucoma suspects in line with the preferential vulnerability of the inferior nerve fibres in glaucoma. Quadrant melanopsin pupillometry provides a linear functional correlate of structural retinal nerve fibre thinning in glaucoma suspects and early glaucoma patients, with potentially excellent diagnostic accuracy in the latter. It may have future applications as a non-invasive and objective clinical tool for monitoring functional changes in melanopsin expressing ipRGCs during disease progression, and detecting functional pupillometric changes in suspects prior to the onset of perimetric deficits.

## Additional Information

**How to cite this article**: Adhikari, P. *et al*. Quadrant Field Pupillometry Detects Melanopsin Dysfunction in Glaucoma Suspects and Early Glaucoma. *Sci. Rep.* **6**, 33373; doi: 10.1038/srep33373 (2016).

## Figures and Tables

**Table 1:** Definitions for the PLR metrics during light stimulation and PIPR metrics after light offset (following Adhikari *et al*. 39).

Metrics	Definitions and Units
Baseline pupil diameter	Average over 10 s pre-stimulus period (mm, %)
*PLR Metrics*	
Transient PLR	Peak % constriction from 180–500 ms after light onset^31^,^43^
Peak pupil constriction	Minimum pupil size during light stimulation (% baseline)
Time to peak constriction	Time to peak constriction amplitude, s^39^
Phase amplitude percentage (PAP)	% difference in the peak-to-trough amplitude between 465 nm and 658 nm sine wave stimuli^32^,^34^
*PIPR Metrics*	
6 s PIPR amplitude	Pupil size at 6 s after light offset (% baseline)^23^,^29^,^46^
Plateau PIPR amplitude	Plateau of exponential model fit to the post-stimulus pupil data (% baseline)^23^

**Table 2:** Clinical characteristics (mean ± SD) of controls, glaucoma suspects, and glaucoma patients.

Characteristics	Control (n = 21)	Suspect (n = 12)	Early (n = 22)	Late (Moderate + Advanced) (n = 12)	p-value
Age (yrs); Gender	58.2 ± 9.2; 3 F, 18 M	61.7 ± 9.7; 5 F, 7 M	66.6 ± 10.6* 11 F, 11 M	69.0 ± 9.1* 6 F, 6 M	0.008
Visual Acuity (logMAR)	0.01 ± 0.02	0.04 ± 0.08	0.07 ± 0.10*	0.07 ± 0.09*	0.03
Contrast Sensitivity	1.67 ± 0.10	1.63 ± 0.10	1.63 ± 0.14	1.54 ± 0.15	0.14
IOL	1 Y, 20 N	2 Y, 10 N	5 Y, 17 N	4 Y, 8 N	—
RNFL Thickness (μm)	86.8 ± 13.7	74.7 ± 10.1	71.9 ± 11.3*	63.5 ± 10.6*	0.0008
VF MD (dB)	−0.61 ± 1.57	−2.20 ± 1.13	−2.71 ± 0.98*	−11.58 ± 3.51*	<0.0001

F, female; M, male; IOL, intraocular lens; Y, yes; N, no; RNFL, retinal nerve fibre layer; VF MD, visual field mean deviation.

*Statistically significant difference to controls.

**Table 3:** Sensitivity and specificity of the superonasal post-illumination pupil response (PIPR) in glaucoma.

	PIPR Cut Off (%)	Sensitivity		Specificity		Positive Likelihood Ratio	Negative Likelihood Ratio
		%	95% CL*	%	95% CL		
Suspect	25.6	91.7	61.5 to 99.8	55.0	31.5 to 76.9	2.04	0.15
Early	17.0	95.5	77.2 to 99.9	75.0	50.9 to 91.3	3.82	0.06
Late	13.8	91.7	61.5 to 99.8	90.0	68.3 to 98.8	9.17	0.09

*CL, confidence limits.
